# Filling the gap between theory and practice: A pilot study on parents' perceptions of integrated care for patients with borderline personality disorder

**DOI:** 10.1002/hpm.3307

**Published:** 2021-08-30

**Authors:** Irene Gabutti

**Affiliations:** ^1^ Department of Management Università Cattolica del Sacro Cuore Rome Italy

**Keywords:** accessibility, clinical pathways, guideline implementation, Italy, psychiatry, stepped care

## Abstract

Clinical pathways are known to be key in managing chronic conditions in an effective and sustainable way. This is particularly true in psychiatry, characterized by chronicity and managerial challenges. In particular, the borderline personality disorder is a highly complicated disorder to manage. Although numerous international guidelines converge on the urge of defining clinical pathways and a stepped‐care model for its effective treatment, it is unknown to what extent these guidelines have been implemented into concrete changes in the provision of care. The objective of this study is to pursue a preliminary assessment of whether there exists coherence between the provision of formal health dispositions or guidelines and end‐users’ perception of the change they should imply. A pilot study investigating the perception of parents of patients with borderline disorder on continuity of care has been conducted in three Italian regions. Results suggest that pathways do exist and are partially able to reach their intended effects, although concrete accessibility is still a major issue. Future studies should build on these preliminary results through quantitative investigation and further explore their causes.

## 1 INTRODUCTION

Health care systems (HCSs) across the world are called more and more to face and treat multi‐pathological patients with chronic conditions.^1,2^ In practical terms, this means that part of their traditional focus on treating diseases in their acute phases–that is in settings such as hospitals–should shift towards a more organised management of diseases also in primary healthcare settings, on an ongoing basis.^3^ ^,^ ^4^ Recurring to ‘inappropriate settings’, in turn, leads to a suboptimal use of the HCS’ (usually scarce) financial resources and–ultimately–produces waste and suboptimal results.^5^

A particular medical field in which chronicity constitutes a highly relevant share of cases is psychiatry.^6^ Although psychiatric disorders are frequent and costly both in monetary and in social terms,^7^ many HCSs seem to struggle in building structured clinical pathways to properly integrate settings for acute and non‐acute phases of the diseases.^8^ Yet, such integrated clinical pathways are now considered key in the provision of effective and sustainable care.^9^

In the broad range of psychiatric disorders, a particularly costly and difficult to manage one is the borderline personality disorder (BPD), historically characterized by a very strong social impact opposed to a factual difficulty in accessing adequate services and treatments.^10^ Many initiatives have been carried out internationally to face the challenge of providing integrated and continuous care in appropriate settings for BPD patients.^11^, ^12^ Nevertheless, a clear understanding of the extent to which this challenge has been successfully implemented seems to be missing. This is a relevant problem both due to ethical and social implications of the disease as well as to implications for healthcare organisations' overall accountability and sustainability.^13^ This study provides a pilot investigation through a qualitative approach on the perception of parents of patients with BPD on the concrete continuity of care provided within three Italian regions. The objective of this work is to provide a preliminary assessment of whether there exists coherence between the provision of formal health dispositions or guidelines and end‐users’ perception of the change they should imply.

## 2 THEORETICAL CONTEXT

The Borderline Personality Disorder is a mental health disorder that includes self‐image issues, difficulty managing emotions and behaviour, and a pattern of unstable relationships.^11^ It usually begins by early adulthood and tends to evolve through the different stages of life.^12^ BPD is so challenging to manage because of the relevant functional disabilities it causes in the long term, being usually associated to harmful behaviours such as substance abuse, depression and eating disorders. Suicide is the major cause of death among patients with this pathology.^14^

Although patients with BPD tend to require more medical prescriptions and clinical service than patients with other mental pathologies,^10^ hence resulting in higher costs for the system,^15^, ^16^ it is very difficult to measure the disorder's monetary and social costs due to their frequently rather discontinuous involvement in pathways. This makes it difficult to measure or even detect many further manifestations and effects of the disease.^12^

The psychiatric scientific community has placed a significant emphasis on how to best manage patients with BPD over the past two decades. The guidelines published by the American Psychiatric Association in 2001^11^ represent one of the first steps in this direction. These are based on key recommendations to health managers and providers, and are enriched by numerous subsequent provisions across the world (e.g.^12^, ^17^, ^18^, ^19^). Some of the main recommendations generally provided include the enhancement of^12^:

‐ A swift and timely accessibility of services for patients (*accessibility*).
‐ An active participation of patients, encouraging them to implement defined strategies to face moments of crisis and for the general improvement of their lives (*patient involvement*).
‐ An ‘ability assumption’, considering history and experiences of patients an asset to solve future problems (*ability assumption*).
‐ Coherence and reliability in implementing therapeutic strategies (*strategy coherence*).
‐ Effective team‐work and communication, by clarifying the roles of the various professionals involved in the continuum of care (*role clarity*).
‐ Realistic expectancies, proceeding by progressive objectives and emphasising the intermediate results achieved step by step (*optimism*).
‐ Structured clinical pathways for the pathology which determine the appropriate setting of care according to the specific phase of the disease (*appropriate settings and continuity of care*).

In other words, there emerges the need to enhance a truly ‘patient‐centred’ approach,^20^ based on continuity of care across settings, through the so called ‘stepped care model’.^21^ This consists in providing the least invasive intervention in the least expensive setting, being treatment effectiveness equal. This should be done in a climate of clarity, enablement, and optimism. Therefore, patients who commonly experience a new set of caregivers as they progress from acute to subacute care settings should not perceive their pathway as fragmented, messy and defective.^20^ The challenge is even more demanding for a pathology, such as BPD, that implies a high risk of non‐compliance and of dropping out by patients.^16^

The negative consequences of fragmented care are indeed numerous: quality of care, costs faced, patients' satisfaction and general clinical outcomes are all at risk in the absence of structured pathways.^16^ These are to be thought of as ‘*multidisciplinary management tools based on evidence‐based practice for a specific group of patients with a predictable clinical course, in which the different tasks (interventions) by the professionals involved in the patient care are defined, optimized and sequenced […]*’.^22^ Nevertheless, although normative and regulatory efforts have shifted towards a patient‐centred and integrated approach, it still appears rather unclear whether, and to what extent, these transitions have resulted into concrete changes in the provision of health services.^8^

Building on contingency theory,^23^ which postulates that organizations and systems adapt to the external environment and rules, and on decoupling theory,^24^ that postulates that although formal compliance to rules occurs this may *not* translate into concrete changes, it is key to assess the concrete implementation of the main recommendations for the enhancement of effective clinical pathways for BPD. Given that for many psychiatric disorders caregivers (and relatives) play a key role in the overall success of treatment strategies,^24^ it is useful to detect their perception on the concrete presence of such recommendations in their relative's clinical experience.

This study, therefore, investigates the perception of parents of patients with BPD on the presence of the main recommendations of international guidelines within the services provided by the regional public health system in Italy. The choice of focusing this pilot study on BPD is related to the particularly challenging issues related to involving such patients in structured clinical pathways. Patients with BPD are frequently unstable and at times difficult to commit.^11^ At the same time, the degree of distress they (and their families) suffer, as well as the overall social and monetary costs the disease causes, make this pathology very urgent to manage effectively. The decision of assessing parents' perceptions instead of patients' ones is due to two main reasons: in the first place, parents may provide a possibly more rational perspective, being BPD patients subject to strong idealisation or devaluation of professionals.^12^ Parents are possibly very close to patients both physically and emotionally, yet may be able to provide a more detached perception of events. In the second place, the study investigates patients' pathways ever since they were children (e.g., continuity of care between services for children and for adults), and patients themselves may find it difficult to remember and judge events during their childhood or adolescence.

## 3 METHODS

A survey has been created building on the main recommendations of the British National Institute for Clinical Excellence Guidelines,^17^ possibly held to be among the leading guiding documents in this field in Europe.^12^ The survey is articulated in the following sections: General information, Accessibility to services, Patient involvement, Ability assumption, Strategy coherence, Clarity of roles, Trust and optimism, Setting appropriateness and continuity of care, Involvement of relatives and families, Role of public and private providers of care. This work, which is part of a broader study, focuses on the sections Accessibility to services, Patient involvement, Setting appropriateness and continuity of care and Role of public and private providers of care. The first three sections explore the perceived presence of some of the main recommendations in BPD treatment. The last investigates parents' perception on the overall effectiveness of the public system and on the extent to which it must be integrated by other forms of help.

The survey included both multiple‐choice and open‐ended questions and was validated by a panel of experts made up of five professionals (psychiatrists or psychologists) and of five parents of patients with BPD. Full convergence was reached by the panel after the second amendment of the text.

A total of 30 parents with children who are or have recently been assisted by the Italian public healthcare system have been invited to participate to the study. The invitation was made by a member of a private association of parents of children with mental disorders. Parents were selected on the basis of the region in which their child is/has been assisted. This had to be a region in which a clinical pathway to treat BPD was developed. Parents were invited to reply to the survey directly online through Surveymonkey between February and April 2020. Although a presentation letter of the study and all the contact details of the researchers were provided to participants, these were invited directly by the member of the association and were never contacted in the first person by the research team. In this way, privacy and anonymity were guaranteed. Participation, subsequent to the provision of a written consensus, was completely anonymous and on a voluntary basis. Given the nature of the data collected (i.e., perception of managerial characteristics of the provision of care), no further ethical approval was necessary.

## 4 RESULTS

Out of the 30 invited parents, 15 agreed to participate. Respondents belonged to three regions meeting the requirement of having formally introduced a clinical pathway. The sample of this study, therefore, is made of 15 parents of patients with BPD assisted (presently or in the recent past) by the public healthcare system.

When questioned about the setting in which their son/daughter had been diagnosed with BPD for the first time, 47% stated that it was detected by a professional working in a private context, 40% replied within a public primary care setting, 13% within a hospital. 40% of the sample declared that no previous mistaken diagnosis was provided before BDP. The remaining 60% stated that misdiagnoses such as bipolar disorder, schizoaffective disorder, psychosis, dissociative disorders were initially made and then disproved. Parents expressed clearly that they experienced a strong lack of continuity of care between paediatric settings and services for adults, with 86% of the sample strongly disagreeing with the statement ‘*The transition between settings has been explained and managed in a fully clear and accurate way*’. The remaining 14% stated it cannot judge, while no respondent agreed with the statement.

**FIGURE 1:**
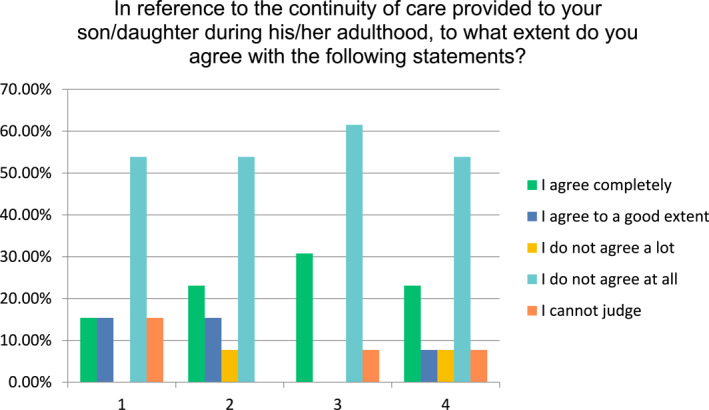
The perception of parents on the continuity of care within clinical pathways. Statement 1: The therapeutic pathway has been conceived in phases, with clear short‐, mid‐ and long‐term objectives; Statement 2: A ‘therapeutic alliance’ aimed at negotiating objectives and a clear activity plan to reach them has been developed and shared with my son/daughter; Statement 3: My son/daughter is or has been supported with social programs aimed at facilitating a job placement; Statement 4: Changes and transitions from one service to another have been prepared and discussed with my son/daughter and/or with me, and have been accurately structured and managed

When questioned about the perception of the clarity of clinical pathways and on the general continuity of care experienced during their children's adulthood, parents expressed a clear perception of a lack of both. In particular, at least 50% of the sample systematically disagreed completely with all the statements aimed at assessing the integration of the pathway's various steps. Figure 1 displays the perceptions of parents on the continuity of care experienced by their son/daughter during adulthood.

The perception of a scarce continuity of care and integration among phases is confirmed by parents' narrative description of their children's experiences. For example, a parent wrote: ‘*We had an excellent experience with child psychiatry services, but the transition to services for adults took place by just ‘passing the buck’ to them. Since then, my daughter has refused to receive assistance*’. Another parent stated that ‘*the local health unit provided just a few clinical visits of 5 to 10 minutes each, and this only after having called 5 or 6 times to insistently ask for them*’.

Forty seven percent of the sample stated that there has been no active involvement of their son/daughter in the definition of his/her clinical pathway, having he/she undergone clinicians' decisions passively. On the contrary, 53% stated that such involvement has occurred, but half of these respondents declared that it was only partially effective.

**FIGURE 2:**
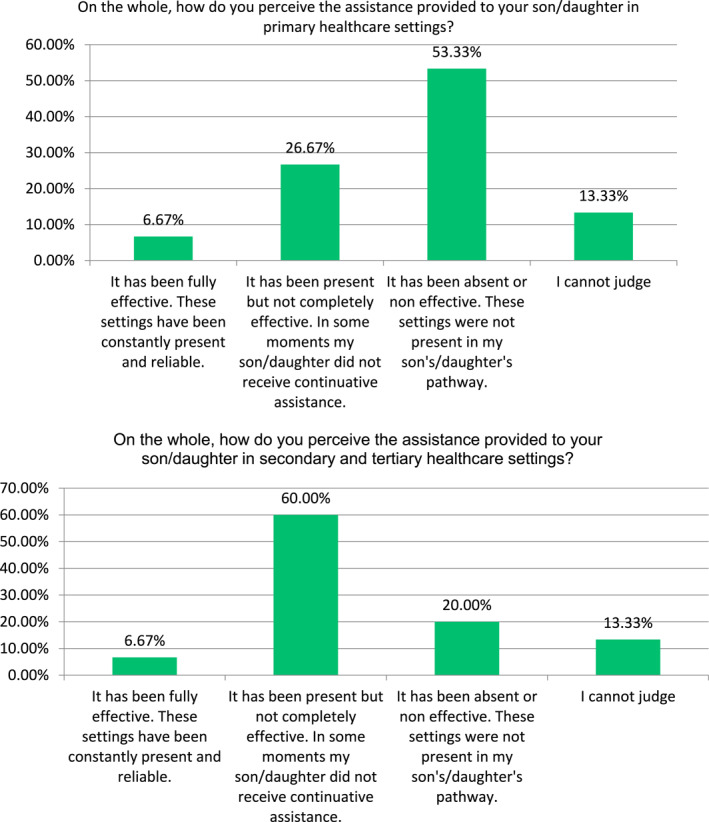
Parents' perception of the effectiveness of provision of care in primary versus secondary and tertiary settings. Source: Author's elaboration

Interesting data emerges from parents' perception on the effectiveness of both primary and secondary care clinical settings. For the first, 53% of the sample declared that they were either absent or non‐effective, while this percentage drops to 20% in reference to secondary healthcare settings (although 60% affirmed that they were only partially effective). Both types of settings were found completely effective and reliable only by 7% of the sample. In Figure 2, the perceptions on the effectiveness of the two types of settings are compared.

Finally, 20% of the sample considers the regional public health system to have been of great support, 60% of relative support although not completely effective and 33% declared it was absent and did not support their child (while 7% stated it cannot judge). For example, a parent said: ‘*We received greater attention only after a suicide attempt and after our firm and insisting requests. Before that, we felt left alone*’. Another parent explained that ‘*although the ‘burden’ of taking care of a relative with BDP is still on families, I think that now providers are working to implement a truly integrated and effective provision of care. Nevertheless, today families receive little psychological and economic* *support* *, and they struggle with the stress of having a child with DPB*’.

## 5 DISCUSSION

This pilot study provides insights on the distance that can occur between advances in scientific evidence and relative guidelines in health management on one hand, and the concrete capacity or possibility for local settings to implement them, on the other. Although the awareness of the urge to conceive care for BPD through a stepped care approach and through an integrated continuum of services is high due to the numerous international directives and guidelines in our HCSs, there seems to exist an important gap between what expected in theory and what concretely perceived by end users (or by their relatives).

The first point of interest is related to the setting in which the disease is detected. Although this preliminary data is encouraging, insofar as it suggests that diagnoses are made in primary healthcare settings a lot more than in hospitals (hence suggesting that they are not made during acute phases of the disease), the very high percentage of diagnoses made in private settings may indicate, if confirmed by future quantitative data, that there persists an actual difficulty in having access to public services. Considering that barriers to accessibility are usually connected to higher social and monetary costs in the long run,^25^ the issue is of both social and economic relevance. Furthermore, there seems to be an intrinsic difficulty in providing a clear diagnosis swiftly, with more than half of the participants stating that misdiagnoses were provided initially. Without addressing issues related to the clinical assessment of possible BPD patients, this data could indeed confirm the weakness of the ‘first steps’ of pathways, which do not support professionals in early BPD detection.

The overall perception of parents is that clinical pathways still appear quite fragmented. The transition from paediatric services to the ones for adults are lived traumatically and as two disjoint moments. The costs related to this weak link are potentially tremendous, given the time and effort needed to reconnect BPD patients to their clinical pathway if they drop out. When questioned about the clearness of the various clinical phases and of their specific objectives, parents seem to be split into two groups. Slightly less than half of the sample states that these were (at least quite) clear and well‐defined; just over half, on the contrary, states that they were not clear at all. This pattern is confirmed for all the questions investigating the perception of continuity of care. The message that seems to emerge is that there *exist* pathways and that professionals' competencies are adequate to ensure the implementation of the main recommendations of guidelines. Nevertheless, these efforts seem to reach only a limited fraction of the population seeking them. More than half of the sample systematically feels ‘abandoned’ by the system and expresses frustration in not being offered a clear strategy for their children.

This evidence can be supported by what reported in Figure 2. Parents seem to lay more trust in secondary or tertiary healthcare settings than in primary ones. This is rather worrying because it suggests that hospitals, which should intervene only during the acute phases of the disease, are rather considered a reference point, perhaps due to the lack of accessibility to primary healthcare settings. This, in turn, suggests that issues in terms of settings' appropriateness might arise. Indeed, these numbers are to be interpreted in the light of the statement of 80% of the sample, which declares that the public system, on the whole, has at least partially supported their son/daughter.

Summarizing, there are possibly three main messages to focus on. The first has to do with the accessibility to services. Although clear guidelines to manage this disease are present and widely known, there still appear to exist barriers to the actual accessibility to services. A strong share of the pilot sample does not feel taken in charge, suggesting that not everyone has a timely access to them. There seems to exist, in fact, an actual difficulty in addressing patients to the right pathway by providing a timely diagnosis. Moreover, the lack of continuity of care between settings for children and adults may further endorse the concrete difficulty of reaching the pathway in the first place.

The second main message is related to the actual suitability of pathways. Here evidence seems mixed. Once ‘the right pathway’ has been reached, its capability of assuring an integrated provision of care is perceived as at least sufficient (or also very good) by slightly less than half of the pilot sample. This, in turn, suggests that many of the recommendations are indeed implemented, at least for those patients who enter the pathway. Again, the feeling is that a relevant share of the sample feels ‘left out’ and cannot, therefore, perceive its intended positive effects.

Finally, the third message is related to the specific roles of different healthcare settings. The fact that hospitals are more frequently perceived as present than primary care settings is potentially alarming. Hospitals should mostly be present in patients' pathways only in sporadic acute events and are not the settings that should be referred to on a usual basis. This may suggest that there are difficulties in building an effective network on the territory, which is truly able to prevent or limit access to hospitals.

## 6 CONCLUSIONS

This study provides a preliminary assessment of the perception of parents of patients with BPD on the continuity of care provided by Italian regional healthcare systems. Continuity of care is by now held crucial in the effective and sustainable management of chronic conditions. Nevertheless, a number of issues may in fact hinder a concrete and complete implementation of these principles.

Although this pilot study provides a snapshot of parents' perceptions on the implementation of clinical pathways, it is important to highlight some of its limitations, as well as to clearly delimit the contribution it can provide. Being a pilot study, its results cannot, of course, be generalised to the whole population of the regions. These results, therefore, cannot be interpreted as a universal understanding of the inter‐regional scenario. The sample is small and may be biased due to the fact that it has been reached through an association of parents. This implies that this specific group may, to some extent, have had different experiences from those of other sub‐groups of the population. Nevertheless, the work has the advantage of providing initial evidence (which is missing in many contexts, to the best of the author's knowledge) to guide strategic agendas in regional and local contexts. Many of the issues addressed in this work are, in fact, likely to be relevant in local health scenarios across countries.

A second limit has to do with the cross‐sectional perspective of the study. Although the choice of involving parents of patients was exactly aimed at detecting reliable data, some of the questions posed concerned events happening over 20 years ago (e.g., those about the transition from paediatric settings to those for adults). This may imply both that respondents' memories may not be completely accurate and also, more importantly, that the regional organisational and managerial scenario may have evolved in the meantime. Yet, if we do not interpret these results as an assessment of the effectiveness of the local provision of care, but rather as an assessment of a ‘starting point’ for future improvement, this data can provide useful guidance in the definition of effective response strategies.

Finally, a further limit may be given by the specific historical moment in which the survey has been administered, which coincides with a lockdown period in Italy due to the COVID‐19 pandemic. This might have caused further stress and sense of isolation in respondents, negatively affecting their replies. Given the unprecedented event, there exists no evidence on whether and how this may have influenced the study.

Future research should address and overcome these limits. It should provide quantitative assessments of representative samples across different local contexts and provide insights on how different healthcare systems have dealt and are dealing with this issue. Moreover, although BPD is a particularly challenging disorder to manage for the reasons mentioned above, the implementation of clinical pathways to guarantee a patient‐centered provision of care is fundamental to manage also other personality disorders and a very vast array of mental and non‐mental diseases.

Another fundamental topic to address in the future is related to the concrete barriers in the implementation of pathways and, more specifically and related to the results of this work, to their accessibility by patients. Even more than implementing pathways, the challenge seems connected to involving patients in them. Therefore, it is important to understand why this happens. If, on one hand, this can in part be due to managerial and organisational issues, it is likely that external and additional barriers play a key role. For example, BPD patients may be particularly difficult to involve and commit due to the manifestations of their disorder. In general, the disease may cause dramatic personal and social issues, making it difficult to build and maintain an integrated role in the society. For example, issues such as not having a job (with its negative economic consequences), of being over‐weight or of making substance abuse, may all in turn cause marginalisation and make it difficult to reach providers of care. Future research should further investigate the crucial role of associations, communities, informal groups and informal providers of care to contribute to the understanding of the support they can provide in this problem. The feeling is that they could be more involved and connected to the official and institutional entities of care, so to overcome these barriers.^26^

Finally, future studies should adopt longitudinal methodologies in order to monitor the perception of parents in time, while local settings implement their strategies. Such perceptions should be triangulated with those of patients themselves and of the various professionals involved in the provision of care.

## CONFLICT OF INTEREST

The author of this article certifies that she has NO affiliations with or involvement in any organisation or entity with any financial or non‐financial interest in the subject matter or materials discussed in this manuscript.

## ETHICAL STATEMENT

The manuscript does not contain sensitive data or experiments using animals. Therefore no specific ethical approval was necessary by the University or other Authorities.

## Data Availability

The data that support the findings of this study are available from the corresponding author upon reasonable request.
